# Prevalence of Erectile Dysfunction and Associated Factors among Diabetic Patients in a Tertiary Hospital: A Cross-sectional Study

**DOI:** 10.4314/ejhs.v34i6.7

**Published:** 2024-11

**Authors:** Desalegn Yayeh Dagnaw, Abenet Tafesse, Fikru Tsehayneh, Hanna Asefa, Teklil Hagos, Getahun Tarekegn, Koricho Sime

**Affiliations:** 1 Department of Internal medicine, College of Health Sciences, Arsi University, Asella, Ethiopia; 2 Department of Neurology, college of health sciences, Addis Ababa University, Addis Ababa, Ethiopia; 3 Department of Internal Medicine, College of Health Sciences, Addis Ababa University, Addis Ababa, Ethiopia

**Keywords:** Ethiopia, Erectile Dysfunction, Diabetes, Prevalence, Determinants

## Abstract

**Background:**

Erectile dysfunction (ED) is one of the earliest chronic complications of diabetes, with a worldwide prevalence ranging from 35% to 90%. This study aimed to assess the current prevalence and related factors of ED in individuals with diabetes.

**Methods:**

An institution-based cross-sectional study was conducted with 269 participants. A validated questionnaire, including the International Index of Erectile Function-5 (IIEF-5), was utilized. Statistical analyses included chi-square tests and logistic regression to calculate the odds ratio (OR) and 95% confidence interval (CI). The strength of the association was determined using odds ratio (OR) with a 95% CI.

**Results:**

The mean age of respondents was 54.45 ± 13.19 years, and the mean duration of diabetes was 12.89 ± 8.98 years. Among the 269 respondents, 243 (90.3%) experienced varying degrees of ED. Statistically significant positive associations were identified for patients with a longer duration of diabetes (adjusted OR [AOR]: 19.10CI: 2.35-155.31), microvascular complications (AOR: 6.75,CI:1.55-29.17), insulin therapy (AOR: 4.76,CI: 1.34-17.07), comorbidities (AOR: 8.77,CI: 3.06-25.17), and medications other than those for diabetes (AOR: 4.62,CI: 1.16-18.45). Notably, 82.2% had never discussed ED with their physicians.

**Conclusion:**

The prevalence of ED among diabetic patients is alarmingly high. This study identified significant positive associations between ED and factors such as diabetes duration, microvascular complications, insulin therapy (with or without oral agents), comorbid conditions, and the use of non-diabetes medications. Therefore, screening, treatment, and addressing key determinants of erectile dysfunction should be integrated into diabetes care.

## Introduction

The number of individuals with diabetes increased from 108 million in 1980 to 422 million in 2014, with projections estimating 629 million by 2045. Sexual function is an important indicator of health and quality of life; thus, sexual dysfunction is closely linked to poor social interactions, psychological well-being, and partner relationships. The psychological impact extends beyond patients with ED, affecting their partners, who may feel guilt, inadequacy, or distance in their relationships.

While epidemiological studies show variable results, most indicate that the prevalence of erectile dysfunction is significantly higher among men with diabetes compared to the general population. Furthermore, men with diabetes may experience ED 10 to 15 years earlier than those without. An estimated 34% to 45% of men with diabetes experience ED globally, with individual studies reporting prevalence rates between 20% and 94.6%. A meta-analysis revealed an overall prevalence of 61.62%, with more recent publications indicating rates as high as 67.29%.

In Ethiopia, published studies are scarce and primarily hospital-based, reporting prevalence rates from 48.7% in Tikur Anbessa Specialized Hospital to 85.5% in Bahir Dar Felege Hiwot Hospital. Factors such as type 2 diabetes, the duration of diabetes, older age, low income, being divorced or widowed, depression, low educational attainment, comorbidities, and physical inactivity are strong determinants of ED among men with diabetes. Complications like neurological, cardiovascular, and kidney issues are also significantly associated with ED.

Erectile dysfunction is often a private matter, with many men reluctant to discuss it with healthcare providers. Research indicates that few Ethiopian men address this issue with their doctors, with many unaware that ED is a complication of diabetes or do not view it as treatable. Despite the impact of ED on quality of life and the rising prevalence of diabetes, recent data on the prevalence and determinants among patients attending the largest endocrinology follow-up clinic in Ethiopia is lacking.

## Methods

**Study setting and period**: This study was conducted at Tikur Anbessa Specialized Hospital, College of Health Sciences, in Addis Ababa, Central Ethiopia. As the largest public tertiary referral and teaching hospital in the country, it sees an average of 1,022 diabetes cases each month. The study took place from May 1 to the end of July 2021, with data collected by a trained nurse at the Endocrine Clinic.

**Study design and sampling**: An institutional-based cross-sectional study was performed. Using the single population proportion formula and data from previous studies, the sample size was calculated to be 269. Systematic random sampling was employed to select diabetic men attending the TASH endocrine clinic. A 1998 study indicated that approximately 48.7% of diabetic men at TASH were affected by ED. According to the Department of Internal Medicine's monthly report, about 1,022 diabetic patients visit the clinic, with 45% being men (approximately 460). Based on this, it was estimated that around 224 diabetic men with ED would visit the clinic monthly. The sample size was calculated using a single population proportion formula with a 5% margin of error and a 95% confidence level.

In total, 269 diabetic men attending the TASH endocrine clinic during the study period were included. It was estimated that 672 diabetic men with ED would visit the clinic over three months, and every third patient on the nurses' register was randomly selected for interview.

**Study participants and materials**: Men aged 18 years and older with diabetes mellitus (DM) who visited the Tikur Anbessa Specialized Hospital (TASH) endocrine clinic during the study period and provided voluntary consent were included in the study. A total of 269 men with diabetes were interviewed over three months.

### Data collection procedure

**Interview**: Trained data collectors administered a structured questionnaire to each eligible participant and measured their height and weight. A validated questionnaire based on the 5-item version of the International Index of Erectile Function (IIEF-5), previously used in studies in Ethiopia, served as the primary measurement tool. All questionnaires were anonymous, and participants were interviewed privately, allowing them to self-administer the IIEF-5 section.

**Electronic medical record retrieval**: The I-Care number of the study participants was used to access their electronic medical records. This provided data on laboratory profiles, comorbidities, and medications, which were documented in the structured questionnaire.

### Study variables

The primary outcome variable was erectile dysfunction. The study aimed to assess the determinants of erectile dysfunction among diabetic patients. Independent variables were categorized into three groups:

**Sociodemographic**: age, sex, marital status, educational status, occupational status, monthly income

**Lifestyle habits**: history of alcohol use, regular physical exercise, history of smoking

**Clinical variables**: type of DM, duration of DM, HbA1c levels, diabetes-related microvascular complications, medications for diabetes, hypertension, chronic kidney disease (other than diabetic kidney disease), ischemic heart disease, peripheral artery disease, stroke, psychiatric conditions, and medications other than those for diabetes.

**Data quality**: The questionnaire was translated into Amharic. The principal investigator supervised the data collectors and reviewed completed questionnaires daily for accuracy and completeness.

**Statistical analysis**: Raw data were collected, entered, stored, and analyzed using SPSS version 25. The chi-square test assessed the significance of associations between categorical variables, while binary logistic regression evaluated the strength of those associations. Adjusted odds ratios with a 95% confidence interval were calculated, and p-values below 0.05 were considered statistically significant.

## Results

**Sociodemographic, lifestyle, and clinical characteristics**: All 269 participants who visited the endocrine clinic at Tikur Anbesa Specialized Hospital during the study period were interviewed, resulting in a response rate of 100%. Table 1 shows the sociodemographic characteristics and lifestyle habits of the study participants. The age of participants ranged from 20 to 77 years, with a mean age of 54.4 ± 13.2 years. The majority (46.5%) were aged 41-60 years, while only 17.8% were under 41 years old. Approximately 78.8% of the participants were married, and nearly half (43.5%) had attained a college education or higher. Government employees comprised 26.8% of respondents, while nearly a quarter (24.9%) were retired. The remaining participants included merchants (23.4%), farmers (7.4%), and others (17.5%).

**Table 1:** Socio-demographic characteristics & lifestyle habits of men with diabetes attending TASH endocrine clinic from May 1 2021 to the end of July 2021, Addis Ababa, Ethiopia (N=269)

Variable		Frequency	Percent
Age in years	20-40	48	17.8
	41-60	125	46.5
	>60	96	35.7
Marital status	Unmarried	38	14.1
	Married	212	78.8
	Others*	19	7.1
Educational level	No formal education	9	3.3
	Primary school	49	18.2
	Secondary school	94	34.9
	College & above	117	43.5
Occupation	Farmer	20	7.4
	Merchant	63	23.4
	Government employee	72	26.8
	Retired	67	24.9
	Others**	47	17.5
Monthly income in ETB	< 1500	80	29.7
	1500 - 5000	126	46.8
	>5000	63	23.4
Alcohol history	Yes	89	33.1
	No	180	66.9
Regular exercise	Yes	219	81.4
	No	50	18.6
Smoking history	Yes	6	2.2
	No	263	97.8
Body mass index	≤24.9	138	51.3
	>24.9	131	48.7

* **Others:** divorced & widowed

** **others:** daily laborer, student, NGO

Most respondents were non-smokers (97.8%) and non-drinkers (66.9%). Nearly three-quarters (76.6%) had a monthly income of less than 5,000 ETB. About 81.4% reported engaging in regular exercise of varying intensities, and approximately 51.3% had a BMI of less than 24.9 kg/m^2^ than three-quarters of participants (77.7%) had type 2 diabetes mellitus (T2DM). Table 2 shows medical characteristics of the study participants. The mean duration since diagnosis was 12.9 ± 9 years, with a range from newly diagnosed to 45 years. A significant proportion of respondents had been diagnosed for 2-10 years (36.1%) or 11-20 years (33.1%). Most participants (43.5%) were on oral hypoglycemic agents only, while 30.5% were on insulin only, and the remaining 26.0% were on both oral agents and insulin.

**Table 2:** Medical characteristics of diabetic men having follow up at TASH endocrine clinic from May 1, 2021 to the end of July 2021(N=269)

Variable		Frequency	Percent
Type of diabetes	T1DM	60	22.3
	T2DM	209	77.7
Diabetes related complications	Yes	95	35.3
	No	174	64.7
Type of diabetes related complication (N=95)	Retinopathy	22	23.2
	Nephropathy	24	25.3
	Neuropathy	33	34.7
	>1 complications	16	16.8
Comorbidity	Yes	192	71.4
	No	77	28.6
Type of comorbidity (N=192)	Hypertension	62	32.0
	Cardiac problems	13	6.7
	>1 comorbidity	87	44.8
	Others***	32	16.5
Drugs other than for diabetes	Yes	212	78.8
	No	57	21.2
Types of drugs other than for diabetes(N=212)	ASA,statin, ACEI	141	65.9
	Beta blockers	28	13.1
	Amitriptyline	34	15.9
	Others****	11	5.1

**** Others: diuretics, HAART, carbamazepine

More than half of the study participants (53.2%) had their HbA1c levels measured within the six months preceding the interview. Among those with HbA1c data, the mean level was 8.9%, with a standard deviation of ±2.4%. Thirty-five percent of respondents reported having one or more diabetes-related microvascular complications. Among these, the majority had peripheral neuropathy (34.7%), while nephropathy, retinopathy, and multiple complications were found in 25.3%, 23.2%, and 16.8% of participants, respectively.

The majority (71.4%) of the study participants had comorbid conditions, with 44.8% having more than one chronic illness. Hypertension was reported as a comorbidity by 32.0% of participants. In addition to diabetes medications, most participants (78.8%) were taking other medications, with the majority (65.9%) using one or more of the following: aspirin, atorvastatin, and enalapril. Amitriptyline and beta-blockers were also included in the medical therapy of 15.9% and 13.1% of respondents, respectively.

**Prevalence of erectile dysfunction shown on table 3**: Among the study participants, a significant majority (90.3%) reported experiencing erectile dysfunction of varying severity. Specifically, 44.5% had mild to moderate erectile dysfunction, as indicated by an IIEF-5 score of 12-16 out of 25. Moderate (8-11) and severe (5-7) erectile dysfunction were reported by 23.5% and 20.6% of respondents, respectively.

**Table 3:** Erectile dysfunction among diabetic men having follow-up at TASH endocrine clinic from May 1, 2021 to the end of July 2021

Variable		Frequency(N)	Percent (%)
Erectile dysfunction	Yes	243	90.3
	No	26	9.7
Severity of erectile dysfunction (N=243)	Mild	27	11.1
	Mild to moderate	109	44.9
	Moderate	57	23.5
	Severe	50	20.6
ED discussion with physician	Yes	48	17.8
	No	221	82.2
Treatment tried for ED	Yes	36	13.4
	No	233	86.6
Knew diabetes ED relation	Yes	179	66.5
	No	90	33.5

Of those experiencing erectile dysfunction, only 17.8% had discussed the issue with their physician, while the remaining 82.2% had never broached the topic. The majority (86.6%) had not sought any treatment for erectile dysfunction. Additionally, nearly 66.5% of respondents acknowledged awareness of a possible relationship between diabetes and erectile dysfunction.

**Determinants of erectile dysfunction shown on table 5**: After conducting multinomial logistic regression, the study found that the duration of diabetes, diabetes-related microvascular complications, and the presence of comorbid illnesses were significantly associated with erectile dysfunction in men with diabetes mellitus. Men who had diabetes for 13 years or longer were found to have a 19-fold increased likelihood of experiencing erectile dysfunction compared to those with diabetes for less than 13 years (AOR: 19.10, 95% CI: 2.35-155.31, P value: 0.001). The presence of one or more diabetes-related microvascular complications was associated with nearly a 7-fold increase in the risk of developing erectile dysfunction compared to those without such complications (AOR: 6.75, 95% CI: 1.55-29.17, P value: 0.011). Additionally, diabetic men with chronic comorbidities such as hypertension, dyslipidemia, or cardiac conditions were nearly 9 times more likely to experience erectile dysfunction than those without these conditions (AOR: 8.77, 95% CI: 3.06-25.17, P value: <0.001).

**Table 5:** Factors associated with erectile dysfunction among diabetic men having follow-up at TASH endocrine clinic from May 1, 2021 to the end of July 2021(N=269)

Variable	COR (95% CI)	P value	AOR (CI 95%)	P value
Above mean age (>54 years)	2.88(1.17-7.09)	0.02	1.96(0.66-5.85)	0.23
History of alcohol	2.219(0.808-6.095)	0.122	2.391(0.661-8.657)	0.184
Occupation	2.741(0.796-9.439)	0.11	0.965 (0.15-6.22)	0.97
Duration of diabetes >13 years	24.80(3.31-185.90)	0.002	19.10* (2.35-155.31)	0.01*
Diabetes-relatedcomplications	4.67(1.36-15.99)	0.014	6.731*(1.55-29.17)	0.011*
Insulin ± oralAgents	3.273(1.369-7.821)	0.008	4.758*(1.327-17.069)	0.017*
Having comorbidity	5.76(2.44-13.60)	<0.001	8.77*(3.06-25.17)	< 0.001*
Drugs other than for diabetes	4.52(1.96-10.42)	<0.001	4.62*(1.16-18.45)	0.03*

COR: Crude odds ratio

AOR: Adjusted odds ratio

* p value <0.05

## Discussion

The study revealed an overall prevalence of erectile dysfunction among men with diabetes of 90.3%, which is significantly higher than a similar study conducted in the same institution 24 years ago. It also exceeds the prevalence rates reported in other studies from Bahr Dar (85.5%), Tigray (69.9%), Jimma (60.4%), Mizan Aman (53.3%), and Egypt (79.4%). The prevalence of erectile dysfunction among diabetic patients aligns more closely with a study conducted in Southeast Nigeria, which reported a rate of 94.7%.

The higher prevalence observed in this study compared to previous reports in Ethiopia may be attributed to differences in baseline characteristics of the respondents, particularly clinical factors, as the study was conducted at one of the largest referral hospitals in the country. In our study, the mean age, mean duration of diabetes, and the proportion of participants with microvascular complications and comorbid conditions were greater than in previous studies. Furthermore, participants were encouraged to self-administer the questionnaire, especially for sensitive questions, which may have contributed to a higher reported prevalence of the issue.

Based on the IIEF-5 erectile dysfunction severity scale, 11.1% of participants had mild ED, 44.9% had mild to moderate ED, 23.5% had moderate ED, and 20.6% had severe ED. Compared to a study conducted in Tigray, severe ED was more prevalent in our study (20.6% vs. 5.2%).

This study highlights that the duration of diabetes, the presence of other chronic medical comorbidities, and diabetes-related microvascular complications are strong predictors of erectile dysfunction, consistent with other similar studies. However, unlike those studies, this research did not find statistically significant associations between erectile dysfunction and advanced age, monthly income, or the use of medications other than those for diabetes.

Of the 243 respondents who reported erectile dysfunction, 82.2% had never discussed the issue with their physician during follow-up. This is similar to a previous report (79.1%) from the same institution over two decades ago. This lack of discussion may stem from the difficulty in establishing rapport with patients, as treating physicians are often rotating internal medicine residents. Furthermore, a majority (77.8%) of participants were waiting for their physicians to initiate discussions about erectile dysfunction, expressing concerns about potential side effects of medications like sildenafil and assuming that erectile dysfunction is an untreatable issue. Only 36 (13.4%) respondents with erectile dysfunction had sought treatment for the problem. Most participants were aware of a possible relationship between diabetes and erectile dysfunction.

In conclusion, the prevalence of erectile dysfunction among diabetic patients is high, with strong associations identified with longer diabetes duration, diabetes-related microvascular complications, and other chronic comorbid conditions. Unfortunately, the condition is often not discussed or treated by physicians. The findings of this study underscore the need for greater attention to erectile dysfunction in diabetic patients during routine medical encounters.

The study was conducted in one of the country's largest referral and specialized hospitals, thus it is possible that the respondents had more complicated medical conditions and comorbidities. This might have contributed to the higher prevalence of ED than previous studies done in other parts of the country. In addition, the study was conducted in a single center which may lead to selection bias.

Frontline healthcare providers should be proactive in assessing & managing erectile dysfunction in men with diabetes. Dealing with erectile dysfunction should be one of the pillars of diabetic education by trained health providers. Further studies should be conducted to assess the impact of ED on the relationships and psychosocial status of diabetic men.

## Figures and Tables

**Figure 1:**
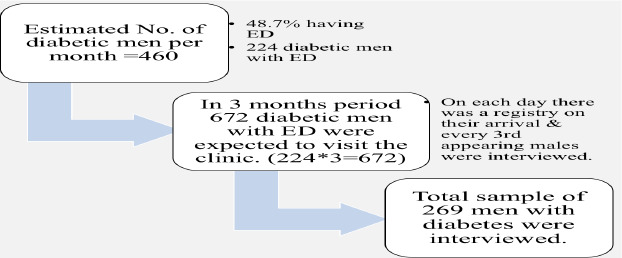
Sampling procedures of diabetic men having to follow up at TASH endocrine clinic May 1, 2021, to end of July 2021

**Table 4:** Comparison of the two studied groups with different clinical parameters

Variable		Respondents with ED	Respondents without ED	P value
Mean age	≤ mean age	118	19	0.017
	>mean age	125	7	
Marital status	Single	52	5	0.797
	Married	191	21	
Occupation	Retired	64	3	0.097
	Non retired	179	23	
Monthly income (ETB)	<5000	192	14	0.004
	≥5000	51	12	
Educational status	< college	140	12	0.263
	≥college	103	14	
History of alcohol	Yes	84	5	0.114
	No	159	21	
History of smoking	Yes	6	0	0.418
	No	237	26	
Regular exercise	Yes	199	20	0.536
	No	44	6	
BMI(Kg/m^2^)	≤24.9	123	15	0.493
	>24.9	120	11	
Type of diabetes	T1DM	53	3	0.165
	T2DM	186	23	
Duration of diabetes	One way	243	26	0.001
	ANOVA			
Mean duration	≤ mean duration	122	25	<0.001
	>mean duration	121	1	
Type of diabetes drug	Oral agents only	99	18	0.005
	Insulin ± oral agents	144	8	
Microvascular complications	Yes	92	3	0.008
	No	151	23	
Comorbidity	Yes	183	9	<0.001
	No	60	17	
Drugs other than for diabetes	Yes	199	13	<0.001
	No	44	13	
